# Establishment of a Direct Competitive ELISA for Camel FGF21 Detection

**DOI:** 10.3390/vetsci12020170

**Published:** 2025-02-14

**Authors:** Yuxuan Yang, Hong Yuan, Yunjuan Jiao, Shuqin Zhao, Yuanfang Fu, Xingwen Bai, Zengjun Lu, Yuan Gao

**Affiliations:** 1 College of Life Science and Technology, Gansu Agricultural University, Lanzhou 730070, China; 2 Lanzhou Veterinary Research Institute, Chinese Academy of Agricultural Sciences, Lanzhou 730046, China

**Keywords:** camel, FGF21, prokaryotic expression, affinity chromatography purification, polyclonal antibody, direct competition ELISA

## Abstract

Camels, with the ability to survive under drought and chronic hunger, developed exceptional efficient lipid reserves and energy substance metabolic characteristics. Fibroblast growth factor (FGF) 21 is a hormone that regulates important metabolic pathways and energy homeostasis. However, the absence of a specific detection method for camel FGF21 impacts research on camels’ metabolic regulation. This study established a direct competition ELISA assay for detecting camel FGF21. Camel FGF21 antigen was expressed and purified through prokaryotic expression system. Polyclonal antibody was produced and purified via immunizing guinea pigs and affinity chromatography assay. Biotin-labeled FGF21 was synthesized artificially as the competitive antigen. After the determination of optimal conditions, including the working concentrations of the antibody and antigen, blocking solution, dilution buffer, and the competition reaction time, the standard curve with a typical “S” shape was generated using GraphPad Prism. The regression equation was Y = 0.1111 + (X^−0.7894^) × (2.162 − 0.1111)/(X^−0.7894^ + 15.76^−0.7894^), with the IC_50_ 15.59 ng/mL, the limit of detection (LOD) 0.024 ng/mL, the limit of quantification (LOQ) 1.861 ng/mL, and the linear range IC_20_~IC_80_ 2.0~119.22 ng/mL. The verification test showed that the recovery rate ranged from 91.34% to 98.9%, and the coefficients of variation for the intra- and inter-plate both were less than 10%, indicating that the ELISA method had high accuracy, good repeatability, and high stability. In addition, this ELISA method had the potential to detect FGF21 secretion levels in other species such as mouse, human, and pig. This study provided a rapid quantitative tool for conducting research on the FGF21 factor in camels.

## Simple Summary

The camel is an amazing domestic species in arid and semi-arid desert regions, with multiple uses such as transportation, milk, meat, racing, and tourism. Camels can store excess fat in their humps, which enables them to survive in drought and eat less for a long period of time. Fibroblast growth factor (FGF) 21 is a key hormone that regulates metabolic pathways and energy homeostasis. However, the absence of a specific detection method for camel FGF21 impacts research on camels’ metabolic regulation. This study has established a direct competition ELISA assay for detecting camel FGF21, which would provide a rapid quantitative tool for conducting research on the FGF21 factor in camels.

## 1. Introduction

The camel is a unique domestic species in arid and semi-arid desert regions, with multiple uses such as transportation, milk, meat, racing, and tourism [1]. It is widely believed that high blood sugar levels and a high salt diet are important adaptive mechanisms for their survival in environments with extremely hot temperatures, sparse vegetation, and limited food and water resources [1,2]. Many metabolism-related genes in camels, including insulin signaling pathway regulation, salt metabolism regulation, and repeated genes crucial for sodium reabsorption, showed faster evolution speed than in other artiodactyls [2]. Moreover, as the main form of energy storage in mammals, fat oxidation could provide energy and water, enabling animals to survive for a long period without eating. Camels can store excess fat in their humps, which enables them to survive in drought and eat less for a long period [3]. Although lipid deposition and mobilization are vital for reproduction, lactation, and environmental adaptation, the knowledge about lipid reserves and application mechanisms of this desert species is still limited.

Fibroblast growth factor 21 (FGF21) was identified as an essential metabolic regulator in 2005 [4]. Unlike the canonical FGF family members’ acting growth stimulating function, such as mitosis, differentiation, and angiogenesis [5], FGF21 exerts as a potent regulator of glucose, lipid, and energy metabolism, but was not observed to be mitogenic [6,7,8]. FGF21 lacks the typical heparan-binding domain that defines the nonendocrine FGFs, which enables its escape from the extracellular matrix into circulation and functions as an endocrine factor [9,10]. In mammals, FGF21 is expressed in many tissues, including the liver, adipocytes, pancreas, muscle, and central nervous system. Specifically, FGF21 is abundantly expressed in the liver and protects it against steatosis via regulating lipid and free fatty acid metabolism in the liver [11]. Additionally, as a promising therapeutic target for metabolism, FGF21 showed the ability to enhance insulin sensitivity, lowing blood glucose and triglyceride (TG) levels, increasing brown adipocyte numbers, preserving β-cell function, inducing sustained weight loss, ameliorating hepatic steatosis, and reducing cardiovascular disease risk [12,13,14,15].

Circulating FGF21 exerts biological functions by binding to the receptor complex, composed of FGF receptor (FGFR) and the co-receptor β-Klotho on the cell surface [16,17]. Reports have demonstrated that serum FGF21 concentration is elevated in obese humans and is closely related to many metabolic disorder syndromes [18]. Moreover, serum level of FGF21 has been suggested as a potential biomarker of many metabolic syndromes, such as type 2 diabetes (T2D), nonalcoholic fatty liver, hyperlipidemia, and cardiovascular diseases [19,20].

Enzyme-linked immunosorbent assay (ELISA) is an efficient, sensitive, and commonly used method for detecting circulating protein factors. The commercial ELISA kits for human or mouse FGF21 detection commonly use the double antibody sandwich method, which is characterized by good specificity and high sensitivity. However, this method has high development costs, high detection costs, and robust species specificity. Presently, there is no available test kit for camel FGF21 protein, which poses difficulties for conducting research related to camel FGF21 factor. This study attempted to develop a rapid quantitative detection method for camel serum FGF21 via establishing a direct competitive ELISA detection method based on camel FGF21 polyclonal antibody by immunizing guinea pigs with prokaryotic expression and purification of camel FGF21 protein, which provided a rapid quantitative tool for conducting research on the FGF21 factor in camels.

## 2. Materials and Methods

### 2.1. Bioinformatics Analysis of FGF21 Protein

Camel, mouse, human, and pig FGF21 gene sequences were obtained from GenBank with accession numbers XM_010996368.1, NM_020013.4, NM_019113.4, and NM_001163410.1, respectively. Signal peptide was analyzed using the SignalP-6.0 network tool (https://services.healthtech.dtu.dk/services/SignalP-6.0/, accessed on 4 November 2022). Sequence alignment was performed with CLUSTALW (https://www.genome.jp/tools-bin/clustalw, accessed on 7 November 2022). Protein spatial structure was predicted using SWISS-MODEL (https://swissmodel.expasy.org/, accessed on 7 November 2022).

### 2.2. Vector Construction and Protein Expression

The FGF21 gene, after removing the signal peptide sequence, was cloned into pET-28a (+) plasmid containing His tag. The recombinant vector was introduced into *Escherichia coli* (*E. coli*) Rosetta (DE3) cells (Sangon Biotech, Shanghai, China) for protein expression induced by isopropyl β-D-Thiogalactoside (IPTG, Solarbio, Beijing, China). High-concentration urea was used to denature and dissolve inclusion body proteins. Protein purification was performed using Ni-NTA affinity chromatography (Genscript, Nanjing, China). Target proteins were eluted from a high-concentration imidazolium buffer, and protein renaturation was performed using gradient refolding buffer. Protein purity and specificity were monitored by SDS-PAGE electrophoresis and western blot, respectively.

### 2.3. Preparation of Polyclonal Antibodies Against Camel FGF21 Protein

Two-month-old guinea pigs with obtained from Experimental Animal Center of Lanzhou Veterinary Research Institute (Lanzhou, China) were employed to generate anti-FGF21 antibodies. Briefly, purified FGF21 protein emulsified with adjuvant Montanide ISA 201 (Seppic, Paris, France) in equal volume was used to produce a vaccine for the immunization of the guinea pigs, with each guinea pig receiving 400 μL (100 μg recombinant protein) intramuscularly on days 0, 21, and 35, respectively. The serum was isolated after cardiac blood collection on day 42. The purification of guinea pig polyclonal antibodies was carried out using the ÄKTA prime protein purification system and HiTrap Protein A HP antibody purification chromatography (Cytiva, Uppsala, Sweden). Protein purity and specificity were monitored using SDS-PAGE electrophoresis and western blot, respectively.

### 2.4. Preparation of the Biotin-Labeled Antigen

The Biotin-conjugated camel FGF21 antigen was generated using a Biotin conjugation kit (Sangon Biotech, Shanghai, China) and purified by dialysis using phosphate buffer solution (PBS). Conjugate specificity was monitored by western blot using a horseradish peroxidase (HRP)-conjugated avidin antibody (Genscript, Nanjing, China).

### 2.5. SDS-PAGE Electrophoresis and Western Blot Assay

Bacterial lysate or purified protein were obtained for SDS-PAGE electrophoresis and western blot detection. Briefly, the protein concentration was determined using a BCA Protein Assay Kit (KeyGen, Nanjing, China). Equal total proteins were separated via 12% SDS-PAGE gel electrophoresis. The protein bands were visualized with Coomassie Brilliant Blue staining (Solarbio, Beijing, China) and decoloration. For the western blot test, gel after electrophoresis was electrotransferred to polyvinylidene difluoride (PVDF) membranes (Millipore, Bedford, MA, USA). After blocking with 10% skimmed milk in TBST for 2 h at room temperature, the membranes were incubated overnight at 4 °C with primary antibodies, including the anti-camel FGF21 polyclonal antibody mentioned above, anti-His tag antibody (CusaBio, Wuhan, China), or anti-human FGF21 antibody (ab171941, Abcam, UK). After three washes with TBST, the membranes were incubated with the corresponding HRP-coupled secondary antibody (CusaBio) for 1 h at 37 °C. Bands were visualized using the Gel Imaging System (Tannon Science & Technology, Shanghai, China).

### 2.6. Establishment of Direct Competition ELISA Method

Polyclonal antibody diluted in 100 μL coating solution was added to each well of the ELISA plate for coating overnight at 4 °C. After washing with PBST 3 times, the plate was blocked with blocking solution of 100 μL in each well at 37 °C for 1 h and then air-dried for later use. We added 50 μL standard protein diluents, with gradient concentrations of 10000, 2000, 400, 80, 16, 3.2, 0.64, and 0.128 ng/mL, or sample to be tested into each well of coating plate together with 50 μL Biotin-camel FGF21 for incubation at 37 °C for 1 h. After washing 3 times with PBST, HRP-conjugated avidin antibody was added into each well and incubated at 37 °C for 30 min. After washing with PBST 5 times, TMB chromogen solution (Beyotime, Beijing, China) was added into each well and maintained for 15 min. After adding stop solution for TMB substrate (Beyotime, Beijing, China) in each well, an OD value of 450 nm wavelength was recorded under the microplate reader. Fitting curves to establish equations of the relationship between the negative logarithm of camel FGF21 antigen content and OD450 value were made using GraphPad Prism 9 software.

The optimization of reaction conditions for direct competition ELISA was performed based on the maximum OD value (OD_max_), half maximal inhibitory concentration (IC_50_), and OD_max_/IC_50_, including determination of the optimal working concentrations of the encapsulated antibodies (12.5, 10, 7.5, 5 μg/mL) and the enzyme-labeled antigens (20, 15, 10, 5 ng/mL) using a checkerboard titration test, optimization of blocking solution conditions (1% BSA, 2% BSA, 1% BSA 1% sucrose, 1% BSA, 5% sucrose in phosphate buffer), optimization of dilution solution for diluting samples and enzyme-labeled antigens (deionized water, PBST, PBS, 1% BSA), exploring the competitive reaction time at 37 °C (45, 60, 75, and 90 min), and dilution ratio of avidin (1:20,000, 1:25,000, 1:30,000, and 1:35,000).

### 2.7. Determination of Sample Recovery

Camel FGF21 antibody standards were diluted to different concentrations (3, 18, 50, 120 ng/mL) for the detection and calculation of the recovery rate using the ELISA method established above with the following formula: Recovery = (A/B) × 100%, where A represents the theoretical added concentration and B represents the actual measured concentration.

The repeatability test of the established ELISA assay was investigated by comparing the IC_50_ within and between the batches, and the stability of this method was judged according to the shift deviation of the reaction curve. For the determination of intra-batch stability, random wells of the same plate were selected to measure the concentrations of standards and calculate the coefficient of variation under the optimal detection conditions. For the determination of inter-batch stability, different batches of coated plates were purchased to measure the concentrations of standards and calculate the coefficient of variation under the optimal detection conditions. The coefficient of variation was calculated as the ratio of the standard deviation to the mean.

### 2.8. High-Performance Liquid Chromatography (HPLC) Validation

An HPLC instrument with a C18 chromatographic column (3 μm, 2.0 mm × 150 mm) was employed to detect the concentrations of camel FGF21 protein samples in the mobile phase of methanol and PBS, with the flow rate 0.6 mL/min, detection wavelength at 280 nm, and injection volume of 20 μL. The ELISA detection results were conducted to be compared with the HPLC results to verify the accuracy and reliability of the ELISA method.

### 2.9. Detection of Reactivity in Species-Specific Reaction

Mouse FGF21, human FGF21, and pig FGF21 protein concentrations were detected using the established ELISA assay. On the original reaction system, a gradient dilution of the three proteins was used as a standard, and an attempt was made to establish a standard curve for detecting the concentration of FGF21 protein in different species.

### 2.10. Statistical Analysis

Data are represented as the mean ± SEM of repeated experiments. Statistical analyses were performed with one-way ANOVA, followed by Fisher’s least significant difference test (Fisher’s LSD) and the independent-samples Student’s t test with SPSS software (Version 20.0; SPSS, Chicago, IL, USA). *p* < 0.05 was considered significant.

## 3. Results

### 3.1. FGF21 Protein Is Highly Conserved in Mammals

**Figure 1:**
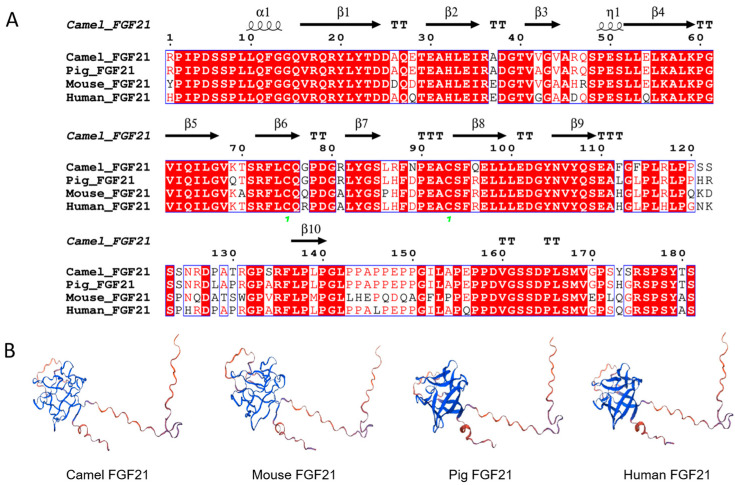
Conservation analysis of FGF21 protein. (**A**) Sequence of camel, human, mouse, and pig FGF21 peptides were aligned with CLUSTALW. (**B**) Spatial structures of camel, human, mouse, and pig FGF21 protein were predicted with SWISS-MODEL, respectively.

To understand the biological characteristics of FGF21 protein, sequence alignment of camel, human, mouse, and pig FGF21 peptides was performed with CLUSTALW, signal peptides were predicted with SignalP, and protein spatial structure was predicted with SWISS-MODEL, respectively. As shown in Figure 1, the FGF21 protein sequence after removing the signal peptide showed high similarity in mammals, including camels, mice, humans, and pigs (Figure 1A). As expected, the FGF21 protein spatial structure is also highly similar, which is composed of a spherical domain rich in β-strands and a long C- terminal random coil (Figure 1B). The result indicated that FGF21 protein is highly conserved, suggesting that FGF21 has important biological functions in mammals.

### 3.2. Expression, Purification, and Validation of Recombinant FGF21 Protein

**Figure 2:**
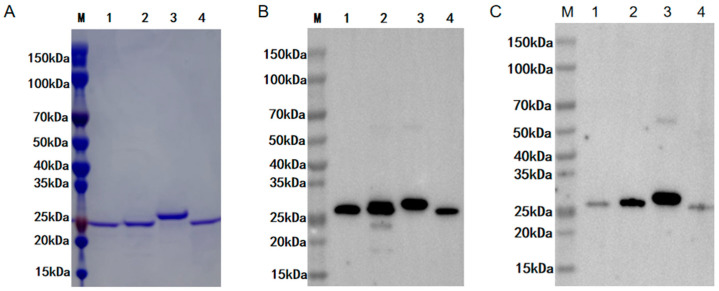
Validation of recombinant FGF21 proteins after purification. (**A**) SDS-PAGE electrophoresis of purified camel, mouse, human, and pig FGF2 proteins. Protein bands were stained with Caumas Brilliant Blue. (**B**) Western blot analysis of purified recombinant proteins using anti-His Tag antibody. (**C**) Western blot analysis of purified recombinant proteins using commercial anti-human FGF21 antibody. M: Protein marker. 1: Recombinant camel FGF21 protein. 2: Recombinant mouse FGF21 protein. 3: Recombinant human FGF21. 4: Recombinant pig FGF21 protein (Supplementary File S1).

To promote research related to camel FGF21 protein, this study constructed FGF21 recombinant vector containing His tag and induced the expression of FGF21 protein in *E. coli*. Protein purification was performed using Ni-NTA affinity chromatography. Protein purity and specificity were monitored using SDS-PAGE electrophoresis and western blot, respectively. As shown in Figure 2, gel electrophoresis determined that this study obtained four high-purity recombinant protein products with a molecular weight of about 25 kD (Figure 2A). Western blot detection using anti-His tag antibody and commercial anti-human FGF21 antibody showed that the purified products are recombinant FGF21 protein with His tag, including recombinant camel, mouse, human, and pig FGF21 protein (Figure 2B,C). Notably, commercial anti-human FGF21 antibody showed high affinity for human and mouse FGF21 protein but low affinity for camel and pig FGF21 protein (Figure 2C). These results demonstrated that this study obtained high-purity camel, mouse, human, and pig FGF21 recombinant proteins, and FGF21 antibodies have species specificity, indicating that it is of great necessity to prepare camel FGF21 antibody for the development of camel FGF21 detection methods.

### 3.3. Preparation of Guinea-Pig-Derived Camel FGF21 Polyclonal Antibody

**Figure 3:**
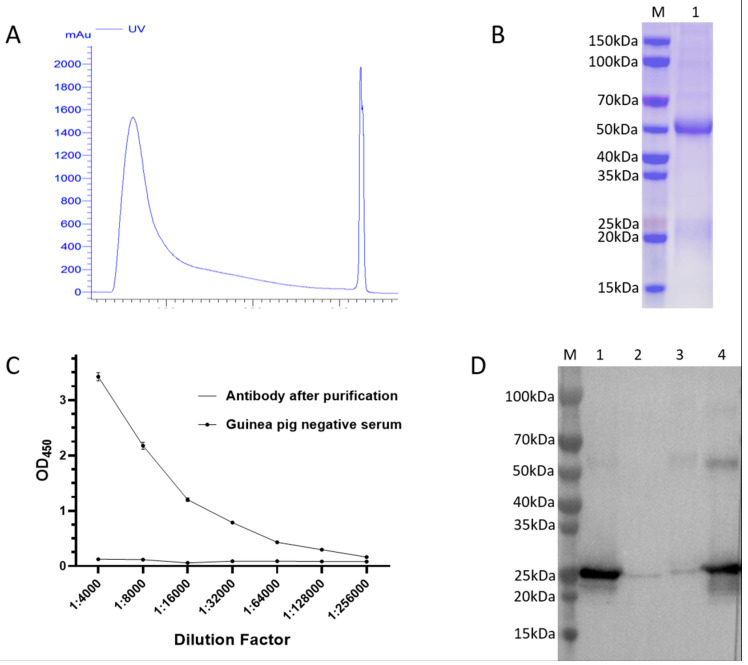
Purification and analysis of guinea pig anti-camel FGF21 polyclonal antibody. (**A**) UV profile during purification of guinea pig IgG using Protein A HP affinity chromatography. (**B**) SDS-PAGE analysis of purified guinea pig IgG. M: Protein marker. 1: Purified guinea pig IgG. (**C**) Antibody titer was determined by indirect ELISA mediated by camel FGF21-encapsulated antigen and HRP-labeled goat anti-guinea-pig IgG. (**D**) Antibody specificity was monitored using western blot. M: Protein marker. 1: Camel FGF21 protein. 2: Mouse FGF21 protein. 3: Human FGF21 protein. 4: Pig FGF21 protein (Supplementary File S1).

To prepare specific camel FGF21 antibody, guinea pigs were intramuscularly immunized with purified recombinant camel FGF21 protein emulsified with adjuvant on days 0, 21, and 35, respectively. Guinea pigs’ serum was collected on 42 d post-immunization for antibody purification using Protein A HP affinity chromatography column. Purified antibody was analyzed by SDS-PAGE electrophoresis. Antibody titer was determined by indirect ELISA mediated by camel FGF21 antigen and HRP-labeled goat anti-guinea-pig IgG, and antibody specificity was monitored with western blot. As shown in Figure 3, only one elution peak appeared during the antibody affinity chromatography purification (Figure 3A). In addition, gel electrophoresis analysis showed that two bands with molecular weights of about 50 kD and 25 kD, corresponding to the heavy chain and light chain of IgG, were clearly visible (Figure 3B), which demonstrated that high-purity antibody IgG was obtained in this study. The indirect ELISA result confirmed that purified guinea pig anti-camel FGF21 polyclonal antibody can react with antigen protein, and the effective dilution can reach 1:12 800 (Figure 3C), indicating that this study obtained high-level production of antibody via the immunization of guinea pigs with purified camel FGF21 recombinant protein. To further determine the species specificity of the antibodies, purified guinea pig IgG was conducted to hybridize with different species’ FGF21 protein. As shown in Figure 3D, guinea pig IgG showed a strong affinity with camel and pig FGF21 protein, but a low affinity with human and mouse FGF21 protein, further confirming that FGF21 antibodies have species specificity. All of these results have demonstrated that high-purity and high-specificity guinea pig anti-camel FGF21 polyclonal antibody was obtained in this study.

### 3.4. Preparation and Validation of Biotin-Labeled Antigen

**Figure 4:**
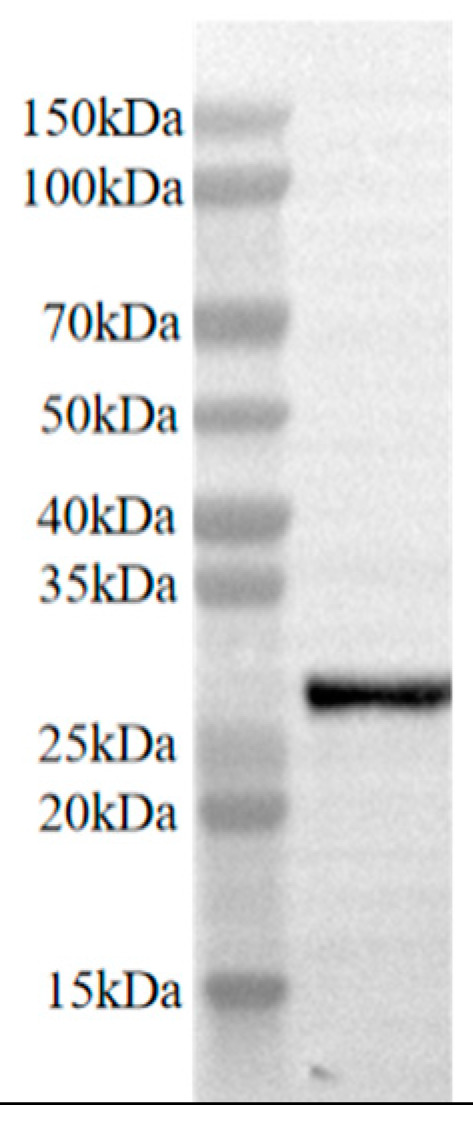
Validation of Biotin-labeled antigen (Supplementary File S1).

To assist in establishing an ELISA detection method for camel FGF21 factor, Biotin-labeled camel FGF21 antigen was generated using a Biotin conjugation kit and monitored with HRP-conjugated Biotin antibody-mediated western blot. As shown in Figure 4, an expected single band above 25 kD was observed on the membrane, indicating that the camel FGF21 protein was successfully labeled with the Biotin tag.

### 3.5. Optimization of Direct Competition ELISA Reaction Conditions

The determination of optimal conditions is crucial for the establishment of ELISA detection methods. To establish a direct competition ELISA detection method for camel FGF21 factor, the maximum OD value (OD_max_), IC_50_, and OD_max_/IC_50_ were used as reference indexes to optimize the reaction conditions and select the best reaction conditions. IC_50_ is termed as a 50% binding rate of the OD value of wells with added inhibitory substances B/and the OD value of control wells without added inhibitory substances B_0_. The smaller the IC_50_ value is, the stronger the specificity and sensitivity of the reaction.

The chessboard method was employed to determine the optimal working concentration of encapsulated antibody and Biotin-labeled camel FGF21 antigen. As shown in Table 1, under the filtering conditions of a larger OD_max_ value, smaller IC_50_ value, and maximal OD_max_/IC_50_, the optimal encapsulated antibody concentration is 10 μg/mL and the optimal Biotin-labeled antigen concentration is 20 ng/mL.

**Figure 5:**
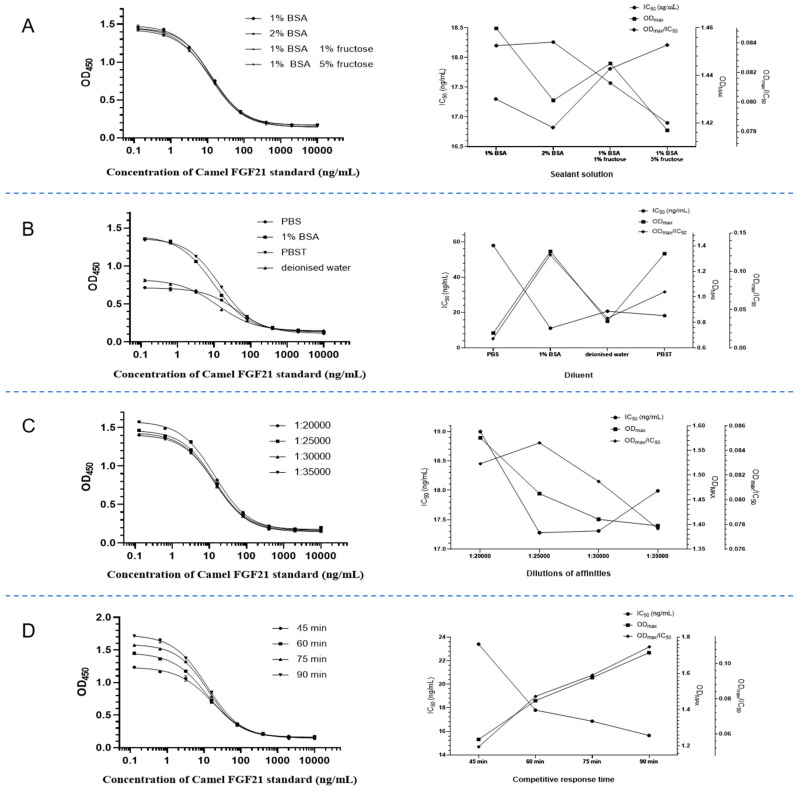
Optimization of direct competition ELISA reaction conditions. (**A**) Screening of the optimal blocking solution. (**B**) Screening of the optimal dilution solution. (**C**) Determination of the optimal dilution factor of avidin. (**D**) Determination of the optimal reaction time.

The blocking solution plays an important role in removing the non-specific binding of antigens and antibodies. Adding sucrose in a blocking solution can reduce the IC_50_ value, due to its role as a protective agent to protect the encapsulated proteins in the ELISA plates, although sucrose has no blocking effect. As shown in Figure 5A, the minimum IC_50_ value and maximum OD_max_/IC_50_ value can be obtained by using a blocking solution containing 1% BSA 5% sucrose in PBS. Therefore, this solution is determined as the blocking solution of the ELISA reaction system.

Dilution buffer is another factor that affects ELISA detection sensitivity. As shown in Figure 5B, dilution buffer of 1% BSA in PBS harvests minimum IC_50_ values and maximum OD_max_/IC_50_ values. Therefore, 1% BSA in PBS was chosen as the standard dilution.

To determine the dilution factor of avidin, the gradient dilution of avidin was performed for OD_450_ detection to calculate the OD_max_/IC_50_ value. As shown in Figure 5C, the maximum OD_max_/IC_50_ value appeared when the dilution ratio of avidin reached 1:25 000, so 1:25000 was considered as the optimal dilution ratio of avidin.

To determine the competition reaction time, different reaction time ranges of 45 to 90 min were performed for OD_450_ detection to calculate the OD_max_/IC_50_ value. As shown in Figure 5D, as the reaction time increases, the IC_50_ continuously decreases and the OD_max_/IC_50_ value continuously increases, indicating that increasing incubation time is beneficial for full competition and makes detection more sensitive. Therefore, 90 min of incubation was selected as the requirement for competition reaction.

### 3.6. Establishment of Standard Curve

**Figure 6:**
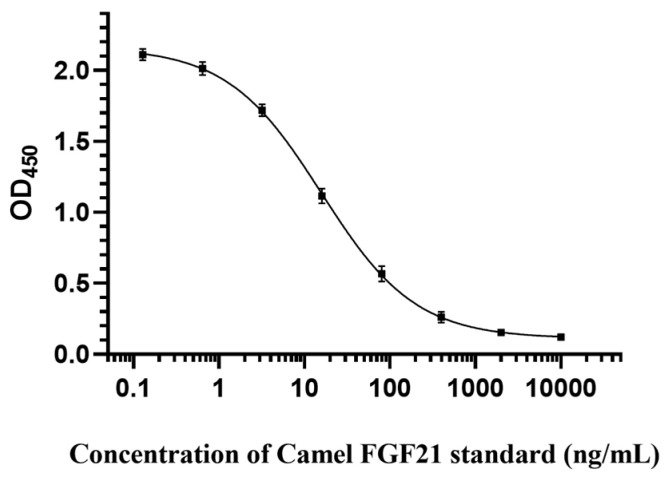
Establishment of camel FGF21 detection standard curve.

Based on the optimal reaction conditions of the direct competitive ELISA method established above, this study generated an “S-type” standard curve (Figure 6). The regression equation is Y = 0.1111 + (X^−0.7894^) × (2.162 − 0.1111)/(X^−0.7894^ + 15.76^−0.7894^), and the correlation coefficient R^2^ is 0.9974.

According to the optimal reaction conditions, this study determined the parameters of the competitive ELISA method by measuring 21 blank samples, finding that IC_50_ is 15.59 ng/mL, the limit of detection (LOD) is 0.024 ng/mL, the limit of quantification (LOQ) is 1.861 ng/mL, and the linear range IC_20_~IC_80_ is 2.0~119.22 ng/mL.

### 3.7. Determination of Recovery Rate

The evaluation of the sample recovery rate is an important indicator for judging whether the ELISA assay is accurate or not [21]. This study used the established direct competitive ELISA method to detect standards of different concentrations and calculate the recovery rate. As shown in Table 2. The recovery rate ranged from 91.34% to 98.9% for four different concentrations of standards, indicating that the detection accuracy of this method is very high.

To further verify the accuracy of the direct competitive ELISA method established in this study, the samples containing camel FGF21 protein were simultaneously detected using HPLC and ELISA. By comparing the HPLC detection values with those from ELISA and calculating the relative deviation (Table 2), it was found that the direct competitive ELISA method established in this study was generally consistent with the HPLC detection results, with a relative deviation of <3.0%, indicating that the ELISA method is reliable and accurate.

This study further evaluated the intra- and inter-batch stability of this method. As shown in Table 3 and Table 4, the standard deviation of the measured sample concentration was small when the concentration ranged from 3 to 120 ng/mL, and the coefficients of variation of the intra-plate duplicate were all less than 10% (Table 3). In addition, the coefficients of inter-batch variation of IC_50_ were also less than 10% (Table 4), indicating that the ELISA method established in this study has good repeatability and high stability, which would meet the detection requirements of field samples.

### 3.8. Species Specificity Verification

**Figure 7:**
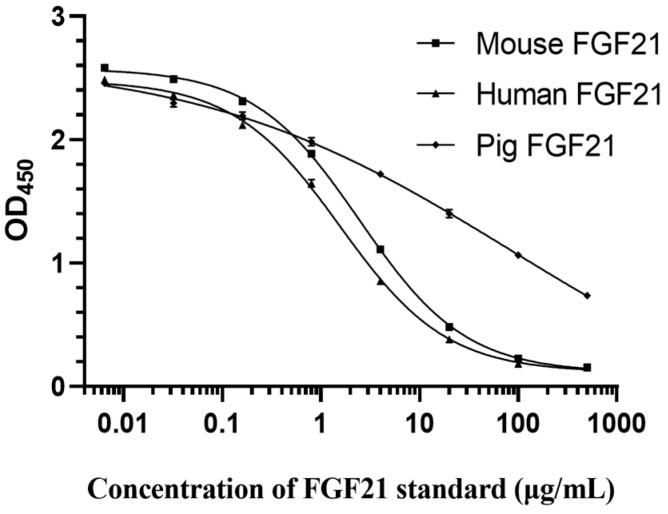
Establishment of mouse, human and pig FGF21 detection standard curve.

To explore the possibility of using this method to detect FGF21 protein concentrations in other species, this study established the standard curves of mouse FGF21, human FGF21, and pig FGF21 based on the optimal reaction conditions. As shown in Figure 7, the standard curves established with mouse and human FGF21 protein were the “S-type”, similar to that of camel FGF21. The regression equations are Y(mouse) = 0.1069 + (X^−0.7974^)× (2.584 − 0.1069)/(X^−0.7974^ + 2.474^−0.7974^), R^2^ = 0.9995; and Y(human) = 0.1111 + (X^−0.7931^) × (2.488 − 0.1111)/(X^−0.7931^ + 1.554^−0.7931^), R^2^ = 0.9983. Intriguingly, the standard curve established by pig FGF21 protein showed a simple decreasing type, with the regression equation of Y = −0.949 + (X^−0.2411^) × (2.682 − 0.949)/(X^−0.2411^ + 269.8^−0.2411^), R^2^ = 0.9988. Notably, all of the R^2^ values were greater than 0.998 in three standard curves, indicating that this ELISA method can also detect samples containing mouse, human, or pig FGF21 proteins with a good degree of confidence.

## 4. Discussion

FGF21 acts as an endocrine modulator in circulation and regulates glucose and lipid metabolism to maintain energy homeostasis in various organizations, especially in the liver, adipose tissues, muscle, and heart, via receptor FGFR1 and co-factor β-Klotho mediated manner [22,23]. The circulating FGF21 level is closely related to many metabolic disorders and is a potential biomarker for many disease [20,24,25]. Moreover, FGF21 is now a promising therapeutic target for metabolic diseases [17,26]. Our previous study revealed that camel FGF21 showed a regulatory pattern distinct from that of humans and mice and exhibited stronger metabolic regulation ability [3,27]. However, the knowledge about the camel-FGF21-mediated regulatory mechanism is still limited, and a specific detection method for the camel FGF21 factor still needs to be established.

ELISA technology is one of the most rapid and sensitive methods for the detection and quantitation of biomolecules using an enzyme-labeled antibody in biomedical research; moreover, as a mature biological detection method, it has the advantages of high sensitivity and specificity [28,29,30]. The ELISA detection method utilizes antigen–antibody reactions to quantitatively or qualitatively detect target substances through enzyme-catalyzed colorimetric reactions. Its application scope covers multiple aspects, such as disease diagnosis, vaccine evaluation, and scientific research. The principle of the direct competition ELISA method is the simultaneous addition of sample antigens (FGF21 protein) and antigens labeled with tags (Biotin-tagged FGF21 protein) to the wells of the microtiter plate to compete for binding to primary antibodies coated on the wells. The more antigens in the sample, the more primary antibodies will bind to the sample antigen. Anti-tag second antibody conjugated to an enzyme is added, followed by a substrate to elicit a chromogenic signal. The concentration of color is inversely proportional to the amount of antigen present in the sample.

Currently, there are few commercial FGF21 protein detection kits, which are developed based on monoclonal antibodies against specific epitopes of human and mouse FGF21 protein and have species limitations. Additionally, high-performance liquid chromatography (HPLC) can accurately analyze the protein concentration in the sample [31]. However, the half-life of the FGF21 protein in vivo is very short. The physical analysis method requires dialysis, purification, ultrafiltration, and other operations on samples, which will affect the accuracy and stability of FGF21 detection. The ELISA method established in this study can complete the detection by only diluting the sample to be tested, which has strong practicability of on-site samples.

This study purified FGF21 protein and prepared guinea-pig-derived primary antibody. Although FGF21 proteins have high conservatism in mammals, anti-camel FGF21 antibody showed low binding affinity to human and mouse FGF21 proteins, and anti-human FGF21 antibody showed low binding affinity to camel and pig FGF21 proteins. However, our data indicated that the ELISA method established in this study can also be used to detect other mammalian FGF21 samples, including mouse, human, and pig. The establishment of direct competitive ELISA methods fills the gap in the research of the detection of camel FGF21 protein, providing a foundation for further research on camel FGF21 protein function.

## 5. Conclusions

This study established a direct competition ELISA assay for detecting camel FGF21 based on artificial Biotin-labeled FGF21 competitive antigen and anti-camel FGF21 polyclonal antibody. After the determination of optimal conditions, including the working concentrations of the antibody and antigen, blocking solution, dilution buffer, and the competition reaction time, the standard curve with a typical “S” shape was generated with the following regression equation: Y = 0.1111 + (X^−0.7894^) × (2.162 − 0.1111)/(X^−0.7894^ + 15.76^−0.7894^). The IC_50_ is 15.59 ng/mL, the LOD is 0.024 ng/mL, the LOQ is 1.861 ng/mL, and the linear range IC_20_~IC_80_ is 2.0~119.22 ng/mL. The verification test showed that the recovery rate ranged from 91.34% to 98.9%, and the coefficients of variation for the intra- and inter-plate were both less than 10%, indicating that the ELISA method had high accuracy, good repeatability, and high stability. In addition, this ELISA method had the potential to detect FGF21 secretion levels in other species such as mice, humans, and pigs.

## Figures and Tables

**Table 1:** Optimal working concentration of encapsulated antibody and Biotin-labeled camel FGF21 antigen.

Enzyme-Labeled Antigen Concentration	Coated Antibody Concentration											
	12.5 μg/mL			10 μg/mL			7.5 μg/mL			5 μg/mL		
	IC50	ODmax	ODmax/IC50	IC50	ODmax	ODmax/IC50	IC50	ODmax	ODmax/IC50	IC50	ODmax	ODmax/IC50
20 ng/mL	24.5	1.73	0.071	20.3	1.617	0.08	22.2	1.362	0.061	22.5	1.49	0.066
15 ng/mL	22.15	1.465	0.066	17.8	1.376	0.077	19.15	1.162	0.061	18.6	1.268	0.068
10 ng/mL	25.5	1.112	0.044	15.1	1.041	0.069	17.2	0.922	0.054	19.7	0.984	0.05
5 ng/mL	37	0.896	0.024	17.5	0.845	0.048	18	0.756	0.042	17.6	0.799	0.045

**Table 2:** Determination of recovery rate.

Theoretical Value (ng/mL)	Average Value (ng/mL)		Recovery Rate		Relative Deviation
	ELISA	HPLC	ELISA	HPLC	
3	2.74 ± 0.20	2.80 ± 0.10	91.33%	93.56%	2.43%
18	17.41 ± 0.81	17.64 ± 0.43	96.72%	98.02%	1.34%
50	49.45 ± 0.74	48.93 ± 0.65	98.90%	97.87%	1.04%
120	116.87 ± 2.38	116.34 ± 0.57	97.39%	96.95%	0.45%

**Table 3:** Intra-batch stability testing.

Theoretical Value (ng/mL)	Average Value (ng/mL)	Coefficient of Variation Within Batches	Coefficient of Variation Between Batches
3	2.71 ± 0.17	6.27%	8.39%
18	17.18 ± 0.9	5.22%	3.25%
50	48.33 ± 0.81	1.67%	2.28%
120	115.67 ± 1.53	1.32%	1.69%

**Table 4:** Inter-batch stability testing.

Batches	IC_50_/(ng/mL)	R^2^
1	15.05	0.9995
2	14.94	0.9944
3	16.3	0.9985
4	15.27	0.9931
5	15.62	0.9971
6	16.1	0.9963
7	15.92	0.9975
Average value	15.6	0.9966
Coefficient of variation	3.52%	0.31%

## Data Availability

All data generated or analyzed during this study are included in this published article.

## Supplementary Materials

The following supporting information can be downloaded at: https://www.mdpi.com/article/10.3390/vetsci12020170/s1, File S1: The original photos of gel electrophoresis and protein blots.
