# Supplementary material for: Establishment of a Direct Competitive ELISA for Camel FGF21 Detection
Source: Vet Sci. 2025 Feb 14;12(2):170. doi: 10.3390/vetsci12020170 (PMC11861717; doi:10.3390/vetsci12020170)
Supplement: Supplementary file 1 [file vetsci-12-00170-s001.zip › vetsci-3462635-supplementary.pdf]

Supplementary materials
File S1: The original photos of gel electrophoresis and protein blots

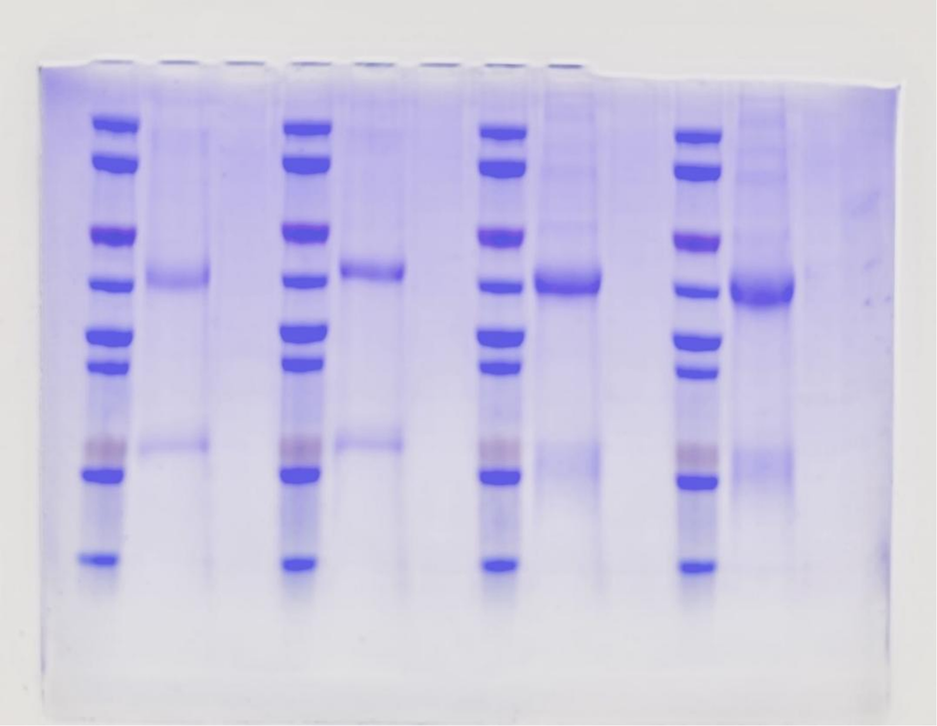

Gel electrophoresis of purified anti-FGF21 IgG

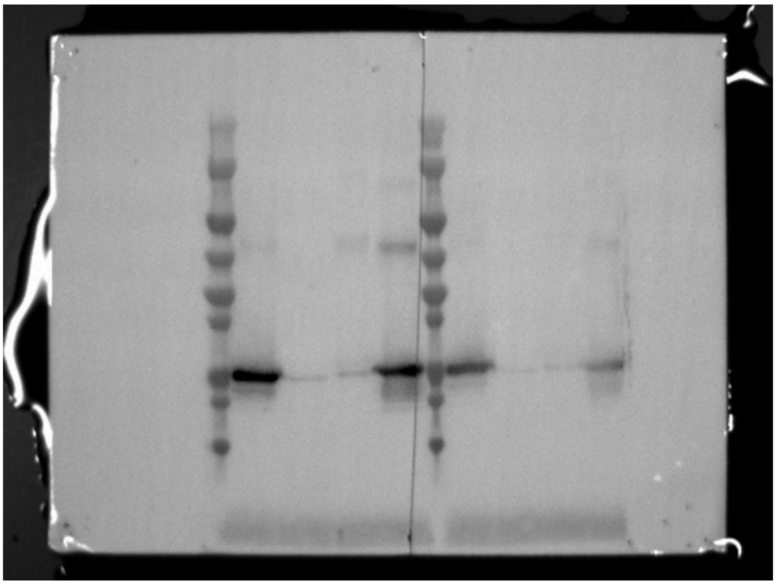

Protein blots of purified anti-FGF21 IgG

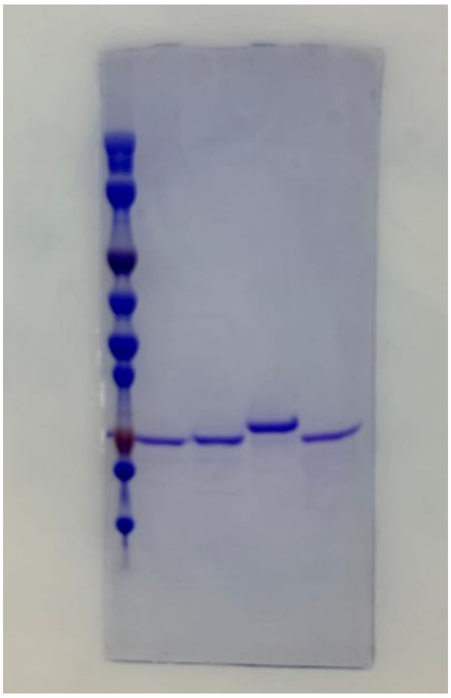

Gel electrophoresis of purified FGF21 protein

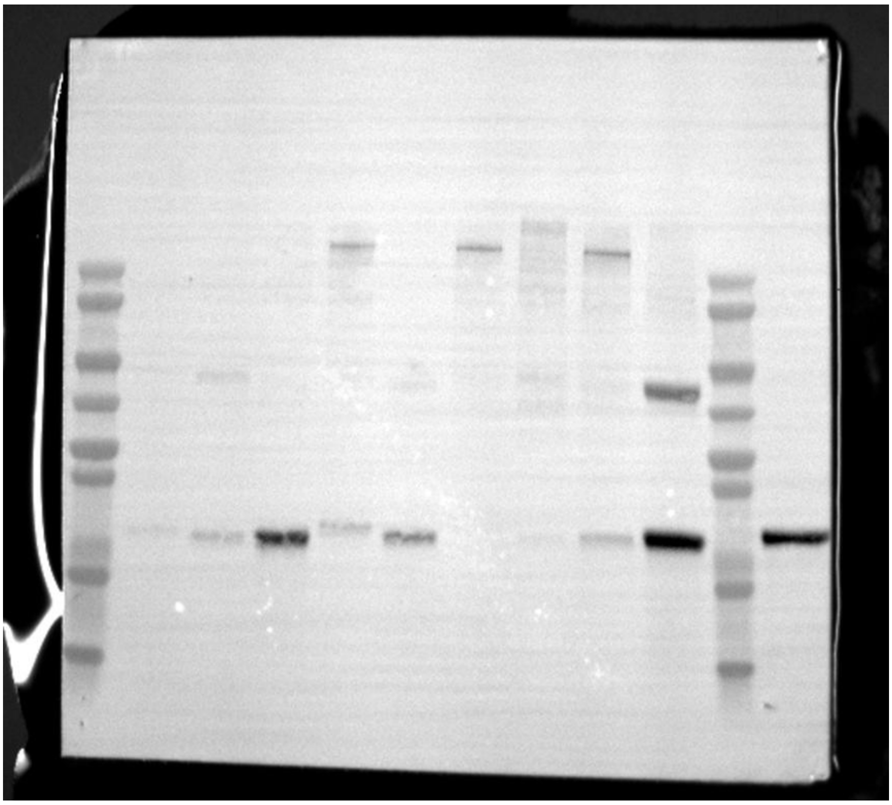

Protein blots of purified Biotin-labeled FGF21

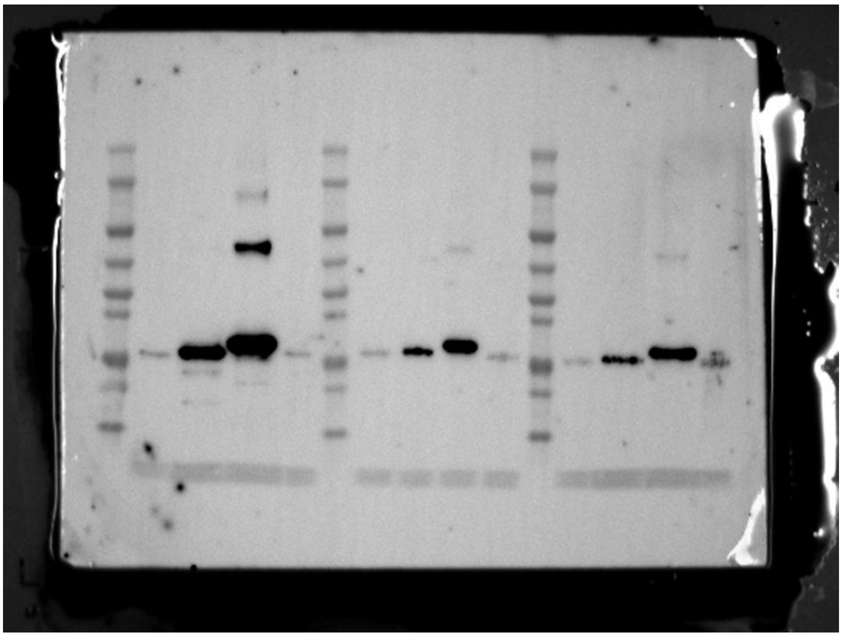

Protein blots of purified FGF21

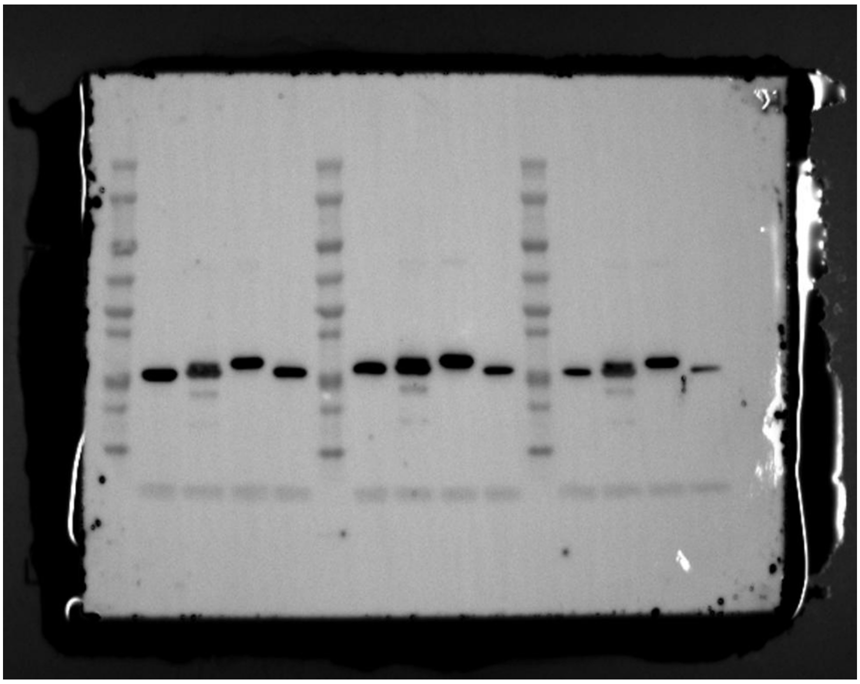

Protein blots of purified FGF21
